# Trends in Alzheimer’s disease mortality in Espírito Santo, Brazil: a time series study

**DOI:** 10.1590/1980-5764-DN-2025-0327

**Published:** 2025-10-24

**Authors:** Ana Luisa Horsth, Marcelo Matieli da Silva, Jéssica Barreto Ribeiro dos Santos, Michael Ruberson Ribeiro da Silva

**Affiliations:** 1 Universidade Federal do Espírito Santo, Centro de Ciências Exatas, Naturais e da Saúde, Grupo de Avaliação, Tecnologia e Economia em Saúde, Alegre ES, Brazil.; 2 Universidade Federal do Espírito Santo, Centro de Ciências Exatas, Naturais e da Saúde, Programa de Pós-Graduação em Assistência Farmacêutica, Alegre ES, Brazil.

**Keywords:** Alzheimer Disease, Mortality, Public Health, Epidemiology, Doença de Alzheimer, Mortalidade, Saúde Pública, Epidemiologia

## Abstract

Alzheimer’s disease (AD) is one of the leading causes of dementia in older adults and is associated with a significant increase in the number of deaths globally.

**Objective:**

This study analyzed mortality from AD in the state of Espírito Santo, Brazil, between 2013 and 2022.

**Methods:**

This ecological time series study used data from the Mortality Information System and the Brazilian Institute of Geography and Statistics, identifying deaths per the International Classification of Diseases 10^th^ Revision (ICD-10) codes for AD (G30, G30.0, G30.1, G30.8, G30.9). Time trend analyses were estimated using Prais-Winsten linear regression to assess annual variations, and the Joinpoint regression model was applied to calculate the annual percentage change (APC).

**Results:**

A total of 6,083 deaths from AD were identified during the study period. We observed a progressive increase in the absolute number of deaths, up from 375 in 2013 to 802 in 2022, as well as in the mortality rate, which rose from 9.77 to 21.02 per 100 thousand inhabitants. We identified a statistically significant upward trend (p<0.001), with a mean annual increase of 49.68 deaths (β_1_=49.68) and an APC of 8.84%. Most deaths were recorded among women, with a mean age of 85.13 years, predominantly among white individuals and widows. Deaths occurred mainly in hospitals, followed by homes.

**Conclusion:**

The study showed a significant increase in mortality from AD in Espírito Santo over the years, reinforcing the importance of preventive strategies aimed at health care and healthy aging.

## RESUMO.

A doença de Alzheimer (DA) é uma das principais causas de demência em idosos e está associada a um aumento significativo no número de mortes em todo o mundo.

**Objetivo:**

Este estudo analisou a mortalidade por DA no estado do Espírito Santo, Brasil, entre 2013 e 2022.

**Métodos:**

Este estudo ecológico de séries temporais utilizou dados do Sistema de Informação sobre Mortalidade e do Instituto Brasileiro de Geografia e Estatística, identificando os óbitos de acordo com os códigos da Classificação Internacional de Doenças 10^ª^ Revisão (CID-10) para DA (G30, G30.0, G30.1, G30.8, G30.9). As análises de tendência temporal foram estimadas usando regressão linear de Prais-Winsten para avaliar as variações anuais, e o modelo de regressão Joinpoint foi aplicado para calcular a variação percentual anual (VPA).

**Resultados:**

Um total de 6.083 óbitos por DA foram identificados durante o período do estudo. Observamos um aumento progressivo no número absoluto de óbitos, passando de 375 em 2013 para 802 em 2022, bem como na taxa de mortalidade, que passou de 9,77 para 21,02 por 100 mil habitantes. Identificamos uma tendência de aumento estatisticamente significativa (p<0,001), com aumento médio anual de 49,68 óbitos (β_1_=49,68) e uma APC de 8,84%. A maioria dos óbitos foi registrada entre mulheres, com média de idade de 85,13 anos, predominantemente entre indivíduos brancos e viúvos. Os óbitos ocorreram principalmente em hospitais, seguidos pelos domicílios.

**Conclusão:**

O estudo mostrou um aumento significativo da mortalidade por DA no Espírito Santo ao longo dos anos, reforçando a importância de estratégias preventivas voltadas para o cuidado à saúde e o envelhecimento saudável.

## INTRODUCTION

 Alzheimer’s disease (AD) is a chronic neurodegenerative disease that affects cognitive functions and is the leading cause of dementia in older adults^1^. AD usually sets in without alarming or severe signs or symptoms. It can develop slowly and over several years^2^. The initial manifestation involves loss of recent memory, disorientation, and difficulties in solving problems, evolving into graver changes such as confusion, mood swings, and inability to perform daily tasks^3^. 

 In 2019, 55 million people worldwide were living with dementia, with AD accounting for 60 to 80% of these cases^4^. According to data from the Global Burden of Disease, deaths from AD increased by 49% between 2009 and 2019, making it the seventh leading cause of death in Brazil^5^. The aging population and the growing prevalence and mortality of the disease underscore the need for cost-effective therapeutic alternatives for its treatment^6,7^ . 

 The diagnosis of AD involves a detailed investigation, including a clinical history of the patient and family members or caregivers, clinical assessment, cognitive tests, laboratory tests and brain imaging^8^. Early detection is essential to enable the immediate onset of treatment, which can be drug and/or non-drug. Although there is no cure, early treatment can slow down the disease’s progression^8,9^. 

 Given the growing impact of AD on public health and the importance of evidence-based decision-making within the Brazilian Unified Health System (SUS), it is essential to understand how mortality data can inform the evaluation and incorporation of effective health interventions. In this context, the National Commission for the Incorporation of Technologies into the Unified Health System — SUS (CONITEC), established by Law No. 12.401/2011, assesses health technologies (HTA) to ensure their efficacy, safety, and cost-effectiveness, guiding decisions to incorporate medicines, products, and procedures into the SUS^ 10,11^. HTA adopts a multidisciplinary approach, considering clinical, economic, ethical, and social aspects, promoting efficiency in the use of resources and evidence-based treatments^12^. 

 In this backdrop, understanding the demographic profile of the target population is crucial. The Brazilian Institute of Geography and Statistics (IBGE) reported that the population of the state of Espírito Santo was 3,833,712 in 2022, of which 631,398 were aged over 60, comprising 284,863 men and 346,535 women, representing 16.47% of the total population^13^. In addition, Espírito Santo ranks sixth in the aging index among the Federative Units, with 58.12%, above the national average of 55.24%^14^. 

 This study aimed to assess mortality from AD in the state of Espírito Santo, Brazil. Research into mortality from AD in Espírito Santo could help formulate more effective public policies to improve the quality of life of older adults. Also, the lack of regional studies makes this topic even more relevant, as it allows local needs to be visualized. 

## METHODS

 We conducted an ecological time series analysis. The inclusion criteria were deaths in the state of Espírito Santo from January 2013 to December 2022 and individuals of all age groups were included. Deaths from Alzheimer’s disease were identified through the underlying cause of death certificates registered in the Mortality Information System (SIM) made available by the Department of Informatics of the Brazilian Unified Health System (DATASUS). The following codes from the International Classification of Diseases 10^th^ Revision (ICD-10) were considered:

- G30—AD
- G30.0—AD with early onset
- G30.1—AD with late onset
- G30.8—other AD
- G30.9—AD, unspecified^15^

There were no exclusion criteria. All deaths due to AD in Espírito Santo were included. 

 The process of data extraction, transforming, and loading (ETL) was carried out using the R programming language and the Microdatasus package, which enables the download and preprocessing of microdata made available by DATASUS. This package automates key steps in the data workflow, including requesting data directly from DATASUS servers, standardizing variables, and converting the data into formats that are more accessible for statistical analysis and data visualization^16^. 

 We adopted the population information from the IBGE to calculate mortality rates, considering the 2010 and 2022 censuses and the intercensal estimates for the other years analyzed. 

 We also characterized the sociodemographic profile of the individuals who died, including variables such as age, sex, region of residence and ethnicity, and identified the main ICDs associated with AD deaths. 

 Socioeconomic indicators, such as the Human Development Index (HDI), the Gini Index, and per capita income, were extracted from IBGE databases and paired with mortality data to enrich the data^17^. This analysis facilitated the assessment of possible relationships between social inequalities and AD deaths. 

 We employed Prais-Winsten generalized linear regression, a model widely used to correct autocorrelation in time series and obtain more robust estimates of trends over time^18^, for the time series analysis of AD-related mortality data. In addition, annual percentage changes (APC) were estimated, and APC trends were identified using Joinpoint regression^19^. 

 We performed data extraction, organization, and analysis through the R programming language in the RStudio development environment, ensuring greater precision and reproducibility of the results. 

## RESULTS

### Profile of individuals who died from Alzheimer’s disease

 A total of 6,083 individuals died from AD. Mean age was 85.65 years (standard deviation [SD] 8.12). Most of these individuals were female (63.21%), widowed (44.06%), and white (60.78%). Most deaths occurred in hospitals (61.30%), followed by homes (30.54%). The municipalities with the highest number of deaths were Vila Velha, Vitória, Serra, Cariacica, and Cachoeiro de Itapemirim. 

 As for the social indicators, the mean Gini index was 0.532 (SD 0.052), the mean HDI was 0.748 (SD 0.057), and the mean per capita income was BRL 977.2 (SD 492.08) (Table 1). 

**Table 1:** Sociodemographic characteristics of individuals who died from Alzheimer’s disease in the state of Espírito Santo, Brazil (2013 to 2022).

Sociodemographic characteristics		Total (n=6,083)
Age in years mean (SD)		85.65 (8.12)
Sex (n, %)		
	Female	3,845 (63.21)
	Male	2,236 (36.76)
	Missing values	2 (0,03)
Race (n, %)		
	White	3,697 (60.78)
	Brown	1,443 (23.72)
	Black	331 (5.44)
	Asian	12 (0.20)
	Indigenous	4 (0.06)
	Missing values	596 (9.80)
Marital status (n, %)		
	Single	530 (8.71)
	Married	1,507 (24.77)
	Widowed	2,680 (44.06)
	Legally separated	221 (3.63)
	Consensual union	23 (0.38)
	Missing values	1,122 (18.44)
Place of death (n, %)		
	Household	1,858 (30.54)
	Hospital	3,729 (61.30)
	Other health establishment	316 (5.19)
	Public roads	22 (0.36)
	Other	153 (2.51)
	Missing values	5 (0.08)
Municipalities with the highest number of deaths (n, %)		
	Vila Velha	959 (15.76)
	Vitória	945 (15.53)
	Serra	563 (9.25)
	Cariacica	525 (7.76)
	Cachoeiro de Itapemirim	360 (5.92)
GINI index – mean (SD)		0.532 (SD–0.052)
HDI – mean (SD)		0.748 (SD–0.057)
Income – average (SD)		977.2 (DP–492.08)

Abbreviations: SD, standard deviation; n, number of individuals; HDI, human development index.

### Main causes of death

 The leading causes of death were unspecified AD (97.48%), late-onset AD (2.35%), other forms of AD (0.11%), and early-onset AD (0.05%) (Table 2). 

**Table 2:** Main causes of death due to Alzheimer’s disease (2013 to 2022).

ICD-10 with the highest number of deaths	Total (n=6,083)
G30.9 – Alzheimer’s disease. Unspecified (n, %)	5,930 (97.48)
G30.1 – Late onset Alzheimer’s disease (n, %)	143 (2.35)
G30.8 – Other forms of Alzheimer’s disease (n, %)	7 (0.11)
G30.0 – Early onset Alzheimer’s disease (n, %)	3 (0.05)

Abbreviation: n, number of individuals.

### Temporal trends in mortality

**Figure 1:**
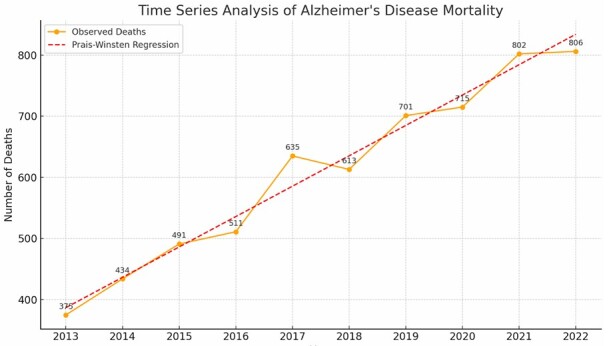
Annual trend in the number of deaths from Alzheimer’s disease in the state of Espírito Santo.

 There was a significant increase in the number of deaths caused by AD in the state of Espírito Santo between 2013 and 2022. In 2013, 375 individuals were registered with one of the ICD-10 codes as the cause of death, while in 2022, this number rose to 802 people (Figure 1). The absolute number of deaths increased in the years observed, except for 2018. The death rate ranged from 9.77 to 21.02 per 100 thousand inhabitants (Table 3 ). 

**Table 3:** Deaths from Alzheimer’s disease in the state of Espírito Santo between 2013 and 2022.

Year	Deaths	APC (%)	Number of inhabitants in Espírito Santo	Death rate per 100,000 inhabitants	Variation
2013	375	-	3,839,366	9.77	-
2014	434	15.73	3,885,049	11.17	+
2015	491	13.13	3,929,911	12.49	+
2016	511	4.07	3,973,697	12.86	+
2017	635	24.27	4,016,356	15.81	+
2018	613	-3.46	3,972,388	15.43	-
2019	701	14.36	4,018,650	17.44	+
2020	715	1.10	4,064,052	17.59	+
2021	802	12.17	4,108,508	19.52	+
2022	806	0.50	3,833,712	21.02	+

Abbreviation: APC, annual percentage change.

 There was an upward trend in the number of deaths due to AD from 2013 to 2022. The estimation coefficient (β1) of 49.68 suggests a continuous increase over the years. In addition, the APC of 8.84% shows that, on average, the number of deaths increased by this proportion each year. Furthermore, the analysis obtained a p-value <0.001, confirming the statistical significance of the results (Table 4). 

**Table 4:** Temporal trends in the number of deaths due to Alzheimer’s disease (2013 to 2022).

Period	Estimate (β1)	p-value	R^2^ (%)	APC (%)	p-value
2013 a 2022	49.68	<0.001	99.84	8.84	<0.001
2013 a 2019	52.11	<0.001	99.76	10.61	<0.001
2019 a 2022	48.16	<0.001	100.00	5.48	0.008

Abbreviation: APC, annual percentage change. Notes: In gray: Prais-Winsten regression; in white: Joinpoint regression.

### Temporal trends in mortality before and during the COVID-19 pandemic

 From 2013 to 2019, we observed a growing trend in the number of deaths due to AD. The estimated coefficient (β_1_) of 52.112 indicates a continuous upward trend over the years. During this period, the APC was 10.61%, meaning the number of deaths increased by this proportion each year. The analysis showed that this trend was statistically significant, with a p-value < 0.001. In contrast, from 2019 to 2022, the number of deaths continued to rise, but at a slower pace. The estimated coefficient (β_1_) was 48.16, and the APC was 5.48%, still reflecting a positive trend, though less pronounced than in the previous period. This trend was also statistically significant, with a p-value of 0.0076 (Table 4 ). 

### Temporal trends of mortality due to Alzheimer’s disease versus all-cause mortality

 A growing trend in the number of deaths from all causes was observed in the state of Espírito Santo. The number of deaths increased from 21,651 in 2013 to 27,992 in 2022, representing a 29.3% growth over the decade. This trend corresponds to an APC of 4.08% (p=0.003) (Supplementary Material - Table S1; Table S2). The death rate per 100 thousand inhabitants also rose over the same period, up from 563.92 in 2013 to 730.15 in 2022 (Table S1). The highest death rate was recorded in 2021, at 798.37 per 100 thousand inhabitants, reflecting the peak of the COVID-19 pandemic (Figure S1). In 2020 and 2021, the number of deaths reached 29,111 and 32,801, respectively, representing the most significant increases in the series: 19.16 and 12.68% compared to the previous years. 

 In contrast, the trend in AD mortality showed a sharper and more consistent increase throughout the same period. While the APC for all-cause mortality was 4.08%, the APC for AD mortality reached 8.84%, with an R^2^ of 99.84% and a highly significant p-value (<0.001), suggesting a strong and linear growth pattern in AD-related deaths (Table 4 ). 

## DISCUSSION

 A total of 6.083 deaths due to AD were identified in the state of Espírito Santo between 2013 and 2022. Most individuals were female, widowed, and white. The mean age was 85.65 years (SD 8.12). Most deaths occurred in hospitals, followed by deaths at home. The municipalities with the highest number of deaths were Vila Velha, Vitória, Serra, Cariacica, and Cachoeiro de Itapemirim. 

 The data analyzed indicate that the advanced age of individuals who died due to AD is consistent with the scientific literature. Studies have shown that the prevalence of AD increases significantly with advancing age, with most cases occurring in individuals aged 80 and over^20,21^. A study conducted by Paschalidis *et al*. (2023), which aimed to analyze mortality trends due to AD in Brazil from 2000 to 2019, found that mean AD mortality rates were considerably higher among individuals aged 80 and above, supporting the findings of the present study^7^. Another study conducted by Piovesan et al. (2023), which aimed to perform an epidemiological analysis of hospitalizations and deaths from AD in Brazil, observed a mortality trend for the same age group^22^. 

 This event is related to pathophysiological processes inherent to aging, such as the progressive accumulation of toxic proteins in the brain. There is an increased buildup of β-amyloid (Aβ) peptide and tau neurofibrillary tangles in brain tissue with age, particularly after the age of 80, leading to neuronal and synaptic loss. Moreover, the glymphatic system, which is responsible for clearing waste from the brain, becomes less efficient with aging, thereby exacerbating Aβ accumulation^23,24^. 

 We observed a predominance of female individuals, which aligns with the literature indicating a higher prevalence of AD among women. This difference may be explained by the longer female life expectancy, often attributed to greater healthcare throughout life^25^. A study conducted within the Brazilian public health system evaluated hospitalizations for dementia and found increases in both short-term hospitalizations and deaths due to AD, particularly among women, thereby reinforcing the findings of the present study^26^. 

 Regarding marital status, most individuals were widowed, likely due to the high mean age of the sample (85.65 years), which increases the likelihood of spousal loss. Widowhood may adversely impact cognitive health, as the spouse often plays an important role in social and cognitive stimulation. The lack of this influence can lead to increased social isolation and physical inactivity, factors that contribute to atrophy in critical brain regions, such as the medial temporal lobe, which is essential for memory^27^. 

 Regarding race, although IBGE data indicate that a large portion of the population in Espírito Santo self-identifies as mixed race brown (49.8%), the analyzed data show that most deaths due to AD occurred among white individuals (60.78%). This contrast highlights the need for further investigations into potential disparities in access to diagnosis and treatment of AD among different racial groups. Additionally, the higher life expectancy observed in white individuals should be considered, as it may contribute to a greater incidence of AD-related deaths within this group compared to others^28^. 

 Socioeconomic indicators also provide relevant insights. The mean Gini index of the individuals who died was 0.532, indicating considerable income inequality within the state. The Gini index ranges from 0 to 1, where values closer to 0 represent greater income equality, and values closer to 1 indicate higher inequality^29^. A study found that the percentage of hospital deaths due to dementia increased in regions with higher and lower economic status. These findings suggest that the results observed in Espírito Santo are not isolated but rather part of a broader national trend^26^. 

 The mean HDI observed in the study was 0.748, which is considered high, given that the HDI ranges from 0 to 1, with values closer to 1 indicating better development conditions^30^. However, the average HDI of Espírito Santo is 0.771, which is slightly higher than the national index of 0.766^31^. 

 The municipalities in Espírito Santo with the highest number of deaths due to AD were Vila Velha, Vitória, Serra, Cariacica, and Cachoeiro de Itapemirim. This pattern may be because these cities have higher population densities and concentrate specialized healthcare services, serving as medical referral centers in the state^32,33^. 

 The mean mortality rate due to AD was 15.31 deaths per 100 thousand inhabitants, with an increasing trend from 2013 to 2022, except for 2018, when a decrease was observed against 2017. National studies support this gradual increase, as observed by Matos *et al*. (2021), who analyzed AD mortality in Brazil between 2010 and 2019, and by Teixeira *et al*. (2015), who identified an upward trend from 2000 to 2009. The mean mortality rates reported by these authors were 35.83 for men and 45.46 for women, considering AD as the underlying cause of death^20, 21^. 

 A growing number of deaths due to AD was observed between 2013 and 2022, up from 375 to 806 cases. However, the rate of increase in AD-related deaths was less pronounced during the COVID-19 pandemic. This could be explained by the possibility that COVID-19 was recorded as the primary cause of death in some cases, potentially leading to an underreporting of AD-related mortality, which could account for the observed findings. International studies indicate that the pandemic significantly affected mortality among patients with AD. A study conducted in the United States found an increase in the number of deaths due to AD and other dementias during the early stages of the pandemic compared to previous years^34^. Moreover, patients with AD were found to be at higher risk of developing severe forms of COVID-19 and of dying from the disease, particularly among older individuals^35^. These findings suggest that the pandemic contributed to increased mortality from dementia, especially among older adults, who are more likely to have comorbid conditions such as heart disease, diabetes mellitus, and other risk factors associated with severe COVID-19 outcomes^36,37^. 

 Finally, our results emphasize that, despite a general increase in mortality in Espírito Santo from 2013 to 2022, the growth of deaths attributable to AD was considerably higher. This trend reflects a mounting burden of neurodegenerative disorders and reinforces the need for targeted strategies in public health planning and care for older people. 

 Given these considerations, this study has some limitations that should be considered when interpreting the results. One relevant limitation is employing population estimates derived from the 2010 and 2022 national censuses, and intercensal projections provided by the IBGE. Although widely used in epidemiological studies, such estimates may be subject to margins of error. Additionally, we should consider the possible underreporting in AD mortality data. Underreporting may occur for several reasons, including diagnostic challenges, inaccurate recording of the underlying cause of death, and limited access to healthcare services in some regions. Another relevant limitation concerns the use of electronic records and secondary data sources. While these allow access to large datasets and facilitate population-level analyses, they are susceptible to missing, inaccurate, or inconsistent information^38^. Furthermore, the COVID-19 pandemic may have influenced the number of AD-related deaths during the study period. 

 In summary, the data reveal a concerning backdrop regarding the rising number of deaths due to AD in Espírito Santo, highlighting the need for more effective public policies aimed at the care of individuals with AD. Future research is essential to delve deeper into the factors associated with this mortality and to support the development of strategies that mitigate the impact of AD on individuals and society. 

 In conclusion, the study shows a significant increase in mortality from AD in Espírito Santo, reinforcing the importance of preventive strategies aimed at the social inequalities observed. Future research should deepen the analysis of socioeconomic conditions and their impact on Alzheimer’s mortality, supporting more effective public policies. 

## Data Availability

The datasets generated and/or analyzed during the current study are available from the corresponding author upon reasonable request.
