# Supplementary material for: Trends in Alzheimer’s disease mortality in Espírito Santo, Brazil: a time series study
Source: Dement Neuropsychol. 2025 Oct 24;19:e20250327. doi: 10.1590/1980-5764-DN-2025-0327 (PMC12561170; doi:10.1590/1980-5764-DN-2025-0327)

**Supplementary Material**

**Table S1.** Deaths from all cases in Espírito Santo, Brazil (2013 and 2022).

| **Year** | **Deaths** | **APC (%)** | **Number of inhabitants in Espírito Santo** | **Death rate per 100,000 inhabitants** | **Variation** |
| --- | --- | --- | --- | --- | --- |
| 2013 | 21.651 | - | 3839366 | 563,92 | - |
| 2014 | 22.030 | 1.75 | 3885049 | 567,05 | + |
| 2015 | 22.332 | 1.37 | 3929911 | 568,26 | + |
| 2016 | 22.868 | 2.40 | 3973697 | 575,48 | + |
| 2017 | 24.112 | 5.44 | 4016356 | 600,35 | + |
| 2018 | 23.500 | -2.54 | 3972388 | 591,58 | - |
| 2019 | 24.431 | 3.96 | 4018650 | 607,94 | + |
| 2020 | 29.111 | 19.16 | 4064052 | 716,30 | + |
| 2021 | 32.801 | 12.68 | 4108508 | 798,37 | + |
| 2022 | 27.992 | -14.66 | 3833712 | 730,15 | - |

Abbreviation: APC, annual percentage change.

**Table S2.** Temporal trends in mortality from all causes in Espírito Santo, Brazil (2013 to 2022).

| **Period** | **Estimate (β1)** | **Value - p** | **R^2^** | **APC (%)** | **Value - p** |
| --- | --- | --- | --- | --- | --- |
| 2013 a 2022 | 1,016 | 0.003 | 66.33% | 4.08 | 0.003 |

Abbreviation: APC: annual percentage change.

Notes: In gray: Prais-Winsten regression; in white: Join point regression.

**Figure S1.** Annual trend in the number of deaths from all causes in Espírito Santo, Brazil (2013 to 2022).


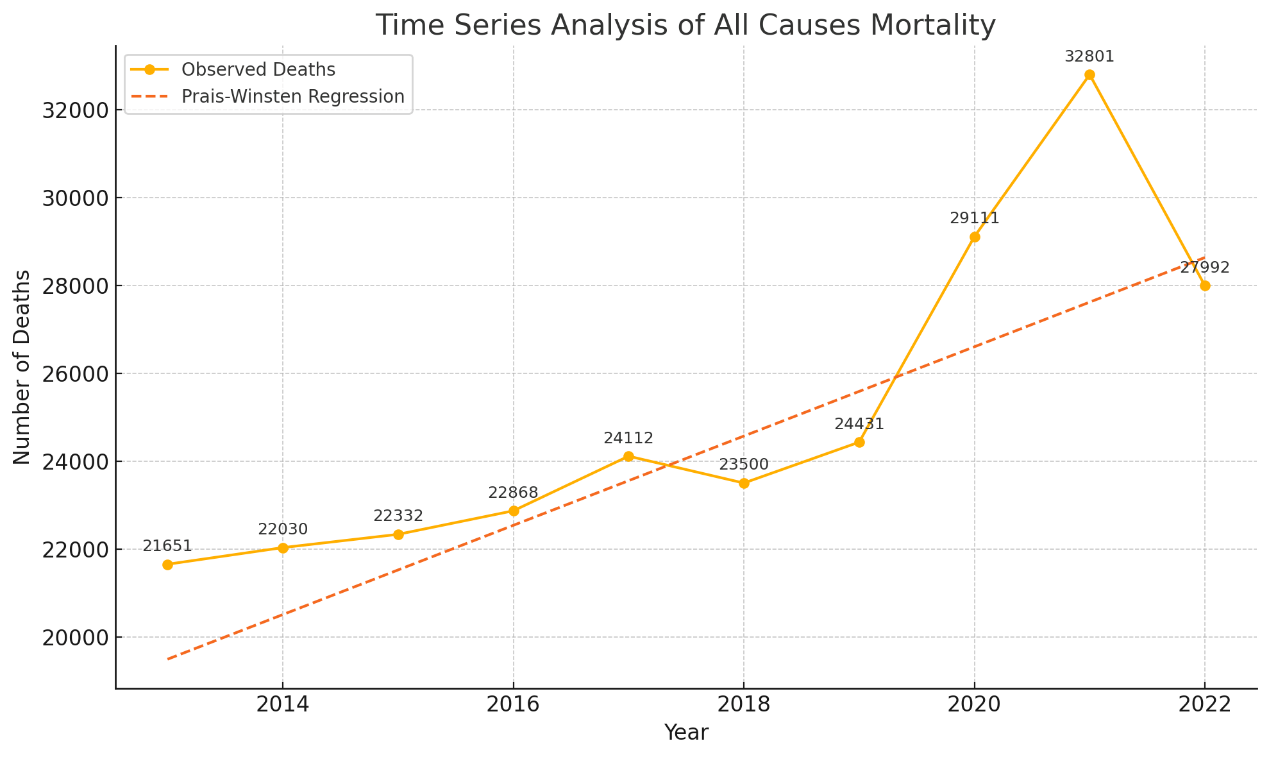

Supplement: Supplementary Table S1 [file 1980-5764-dn-19-e20250327-md1.docx]
